# Epidemiology of Human Anthrax in China, 1955−2014

**DOI:** 10.3201/eid2301.150947

**Published:** 2017-01

**Authors:** Yu Li, Wenwu Yin, Martin Hugh-Jones, Liping Wang, Di Mu, Xiang Ren, Lingjia Zeng, Qiulan Chen, Wei Li, Jianchun Wei, Shengjie Lai, Hang Zhou, Hongjie Yu

**Affiliations:** The University of Hong Kong, Hong Kong, China (Y. Li);; Division of Infectious Disease, Key Laboratory of Surveillance and Early-Warning on Infectious Disease, Chinese Center for Disease Control and Prevention, Beijing, China (Y. Li, W. Yin, L. Wang, D. Mu, X. Ren, L. Zeng, Q. Chen, S. Lai, H. Zhou, H. Yu);; Louisiana State University, Baton Rouge, Louisiana, USA (M. Hugh-Jones);; National Institute for Communicable Disease Control and Prevention, State Key Laboratory for Infectious Disease Prevention and Control, Chinese Center for Disease Control and Prevention, Beijing (W. Li, J. Wei);; School of Public Health, Fudan University, Key Laboratory of Public Health Safety, Ministry of Education, Shanghai, China (H. Yu)

**Keywords:** anthrax, human anthrax, Bacillus anthracis, bacteria, epidemiology, cases, case-fatality rate, bioterrorism and preparedness, respiratory infections, zoonoses, China

## Abstract

Using national surveillance data for 120,111 human anthrax cases recorded during 1955−2014, we analyzed the temporal, seasonal, geographic, and demographic distribution of this disease in China. After 1978, incidence decreased until 2013, when it reached a low of 0.014 cases/100,000 population. The case-fatality rate, cumulatively 3.6% during the study period, has also decreased since 1990. Cases occurred throughout the year, peaking in August. Geographic distribution decreased overall from west to east, but the cumulative number of affected counties increased during 2005−2014. The disease has shifted from industrial to agricultural workers; 86.7% of cases occurred in farmers and herdsmen. Most (97.7%) reported cases were the cutaneous form. Although progress has been made in reducing incidence, this study highlights areas that need improvement. Adequate laboratory diagnosis is lacking; only 7.6% of cases received laboratory confirmation. Geographic expansion of the disease indicates that livestock control programs will be essential in eradicating anthrax.

Anthrax is an acute infectious zoonotic disease caused by the gram-positive, aerobic, nonmotile bacterium *Bacillus anthracis*, which can survive in soil for decades as an extremely resistant form (spores) (*1*). Herbivores become infected when grazing on contaminated land, when bitten by Tabanid flies with contaminated mouthparts, or by ingesting contaminated feed (*2*). Naturally occurring human anthrax infections are caused by contact with infected animals or animal products; ingestion of undercooked infected meat; or exposure to large-scale processing of contaminated hides, wool, and hair in enclosed factory areas (*3*). Injection anthrax has been observed in users of contaminated heroin in western Europe (*4*).

Although there has been a general decrease in the number of anthrax outbreaks in animal populations and in human cases, anthrax still has a nearly worldwide distribution, causes an estimated 20,000–100,000 cases annually, and poses a major public health threat in regions of the Middle East, Africa, central Asia, South America, and Haiti (*5*). In addition, *B. anthracis* is always placed high on the list of potential agents with respect to biologic warfare and bioterrorism because of its robust nature and persistence of spores, the ability of aerosolized spores to readily infect by inhalation, and the high mortality rate for resultant anthrax cases (*6*). *B. anthracis* was used in this context in the anthrax letter events in the United States during 2001 and showed severe consequences (*7*).

Anthrax has probably been present in China for >5,000 years as recorded in ancient Chinese medical books, but few reliable data were available before the People’s Republic of China was founded (*8*). Human anthrax was made a reportable disease in China during the 1950s. Over the past 60 years, great progress has been made in control and prevention, including development of human anthrax vaccine in China during the late 1950s and eradication of anthrax in industrial areas during the 1980s (*8*).

To our knowledge, no published literature systematically describes the epidemiology of human anthrax in China. This study was conducted to observe temporal trends, seasonality, and geographic distribution of human anthrax in China during the past 60 years and demographic characteristics during 2005−2014 to identify the current epidemiologic situation and provide information for control and prevention of this disease.

## Materials and Methods

### National Surveillance Program

The national surveillance program of human anthrax is part of the Chinese Notifiable Disease Reporting System, which was started in the 1950s. During the 1950s–2003, aggregated data for each province (a province in China is similar to a state in the United States) was reported monthly by mail to the Chinese Center for Disease Control and Prevention (China CDC). In 2004, a real-time online nationwide reporting system was implemented. Since then, all human anthrax cases were required to be reported online <2 hours of diagnosis for inhalational anthrax and <24 hours of diagnosis for cutaneous and gastrointestinal anthrax.

### Case Definition

All human anthrax cases, including probable and confirmed cases, were diagnosed according to the unified case definitions issued by the Chinese Ministry of Health. The diagnostic criteria for human anthrax cases changed twice during the study period, in 1998 and again in 2008 (Technical Appendix Table).

Before September 1998, a probable case was defined as a case with clinical manifestations and an appropriate epidemiologic history. A confirmed case was defined as a probable case plus laboratory evidence of *B. anthracis* infection detected by bacterial isolation or demonstration of *B. anthracis* in a clinical specimen by microscopic examination of stained smears.

Since September 1998, a probable case has been defined as a case with clinical manifestations and demonstration of *B. anthracis* in a clinical specimen by microscopic examination of stained smears. A confirmed case is defined as a case with clinical manifestations plus isolation of *B. anthracis* or a >4-fold increase in specific antibody titer against *B. anthracis.*

Epidemiologic history included living in areas with reports of confirmed anthrax or having traveled to such places <14 days before onset or engaging in occupations that are likely to result in exposure to anthrax. The disease has 3 clinical forms: cutaneous, inhalational and gastrointestinal. We have not detected injection anthrax in China.

### Collection and Examination of Specimens

For every suspected anthrax case-patient, appropriate clinical specimens were required to be collected before treatment, including blood for all patients, vesicular fluid for patients with cutaneous anthrax, stools for patients with intestinal anthrax, and sputum or respiratory secretions for patients with inhalational anthrax. Every local CDC (county or prefecture) was required to examine stained smears of clinical specimens by microscopy. The presence of squared-ended, gram-positive, rod-shaped bacteria in chains was considered to be *B. anthracis,* and the corresponding specimens were required to be delivered to the provincial CDC for further bacterial isolation, which was conducted in Biosafety Level 2 laboratories.

In accordance with national surveillance protocol for anthrax (*9*), bacterial isolation was conducted on nutrient agar plates. Specimen were sprayed on plates after preprocessing, which included dilution or sedimentation and centrifugation, and heat-shocking. After incubation for 8–24 hours at 37°C, plates were checked for medium sized bacterial colonies that were hoary and opaque and had a ground-glass–like surface. Phage and penicillin were used to test for sensitivity typical for *B. anthracis*. Because reliable commercial kits for antibody testing are not available, ELISA was rarely used to test for specific antibody against *B. anthracis.* Recently, PCR was also used to provide laboratory evidence of *B. anthracis* infection. However, because there is no unified standard procedure for PCR of *B. anthracis*, PCR was also rarely used in laboratory analysis.

### Demographic, Clinical, and Epidemiologic Data

Human anthrax surveillance data included data for 1955–2014 of probable and confirmed cases for all 31 provinces in China. Aggregated data for cases and associated deaths by province and month were available for 1955–2003. During 2004–2014, each human anthrax case was reported online through a standardized form that included basic demographic information (sex, date of birth, address); occupation; diagnosis classification (probable or confirmed); clinical form (cutaneous, inhalational, or gastrointestinal); outcome (survival or death); date of illness onset; and date of death (if applicable). National population data for China during 1955–2014 were obtained from the National Bureau of Statistics of China (*10*).

### Data Analysis

We included in the analysis all probable and confirmed human anthrax cases with illness onset during 1955–2014. We calculated the annual incidence rate by dividing the number of human anthrax cases by the corresponding population at the end of a given year and the case-fatality rate by dividing the number of human anthrax–associated deaths by the number of human anthrax cases with illness onset by the end of the same year.

We described spread and emergence of human anthrax during 2005–2014 with different kinds of affected counties as follows. A newly affected county this year was defined as a county that reported human anthrax cases for the first time during that year since 2004, a previously affected county with a new case this year was defined as a county that reported human anthrax cases during and before that year since 2004, and a previously affected county without a new case this year was defined as a county that reported human anthrax cases before that year since 2004 but no case in that year. Any county reporting human anthrax cases since 2004 were designated as a previously affected county.

Descriptive statistics included frequency analyses for categorical variables, medians, and interquartile ranges for continuous variables. We used the χ^2^ test for testing differences of proportion for categorical variables. Probabilities were 2-tailed, and p values <0.05 were considered statistically significant. We performed all analyses by using R version 3.0.2 (https://www.r-project.org/) and used ArcGIS version 10.0 (ESRI, Redlands, CA, USA) to plot geographic distribution of cases.

### Ethical Approval

This Chinese National Health and Family Planning Commission (Beijing, China) determined that collection of data for human anthrax cases was part of a continuing public health surveillance. Thus, this study was exempt from institutional review board assessment.

## Results

### Temporal Trend and Seasonality

**Figure 1:**
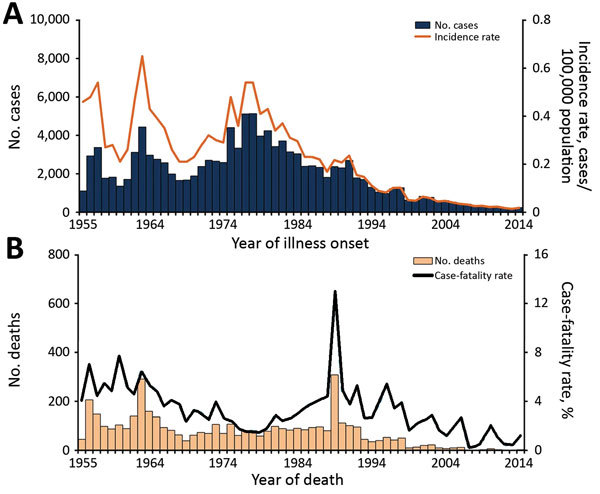
Cases of and deaths from probable and confirmed human anthrax, China, 1955–2014. A) No. human anthrax cases (n = 120,111) and incidence rate (no. cases/100,000 population) by year. B) No. human anthrax deaths (n = 4,341) and case-fatality rate (%) by year.

During 1955–2014, a total of 120,111 probable and confirmed human anthrax cases, including 4,341 fatal cases, were reported to the China CDC; the overall case-fatality rate was 3.6%. Before the 1980s, probable and confirmed human anthrax incidence showed a periodic increase and decrease every 8–10 years. There were 3 major peaks in 1957 (0.54 cases/100,000 population), 1963 (0.65 cases/100,000 population), and 1977–1978 (0.54 cases/100,000 population). Thereafter, incidence decreased until 2013, when it reached a low of 193 cases (0.014 cases/100,000 population) (Figure 1).

**Figure 2:**
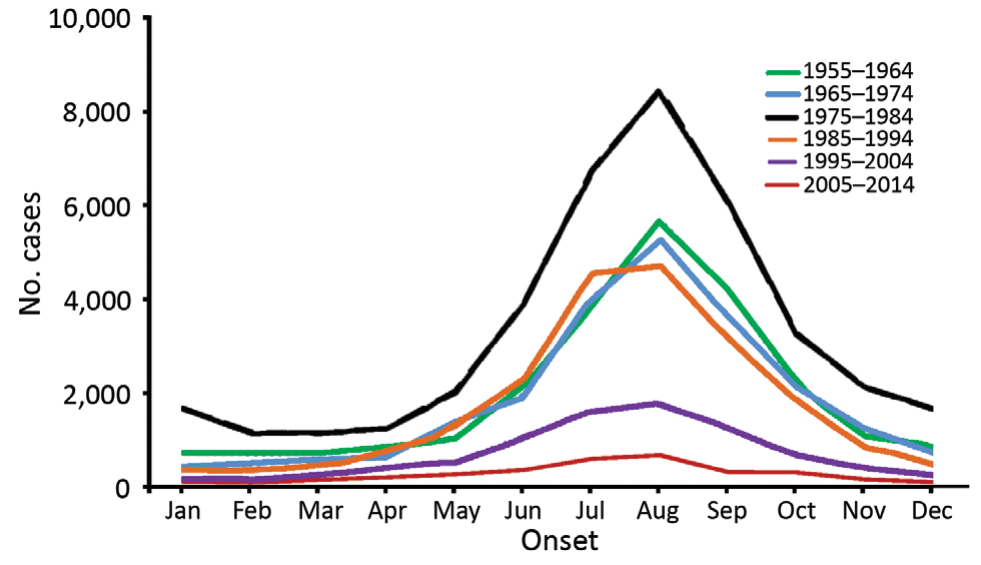
Monthly distribution of probable and confirmed human anthrax cases, China, 1955–2014.

The case-fatality rate showed an overall downward trend before the 1980s and then increased to a high of 13.0% in 1989, which was followed by a generally fluctuating decrease until 2014, when only 3 deaths were reported (Figure 1). Human anthrax cases occurred across the whole year, typically increasing in May, peaking in August (56% of cases occurred during July–September), and decreasing toward November. This pattern was consistent during 1955–2014 (Figure 2).

### Geographic Distribution

**Figure 3:**
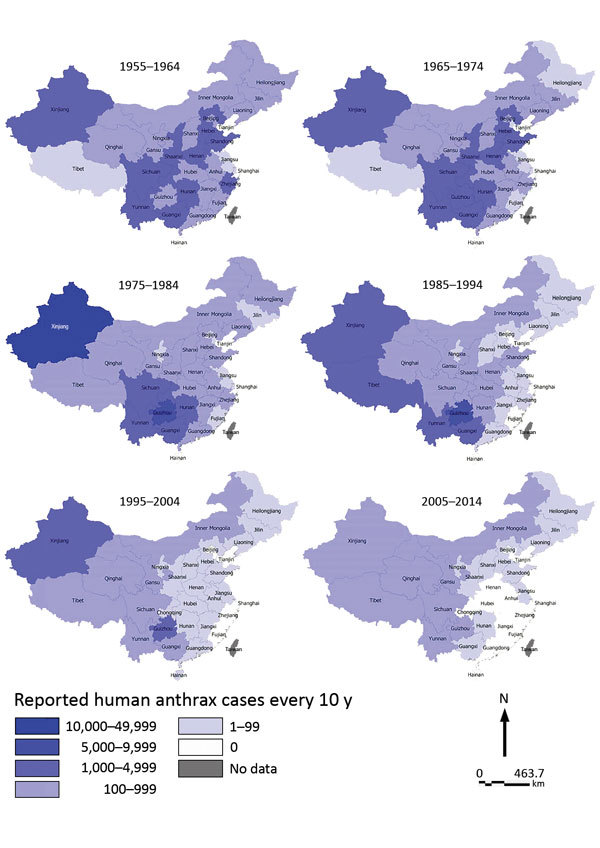
Provincial distribution of probable and confirmed human anthrax cases, China, 1955–2014.

All 31 provinces had >1 probable or confirmed case of human anthrax during 1955–2014. The distribution of cases showed an overall decrease of cases from western to eastern China. From the end of 1970s onward, some large cities, such as Shanghai (1979), Beijing (1984), and Tianjin (1985), and some provinces in eastern China, such as Fujian (1981), Zhejiang (1990), Jiangxi (1996), and Guangdong (1998), gradually stopped reporting human anthrax. In addition, cases were rarely reported in southeastern China (Figure 3).

**Figure 4:**
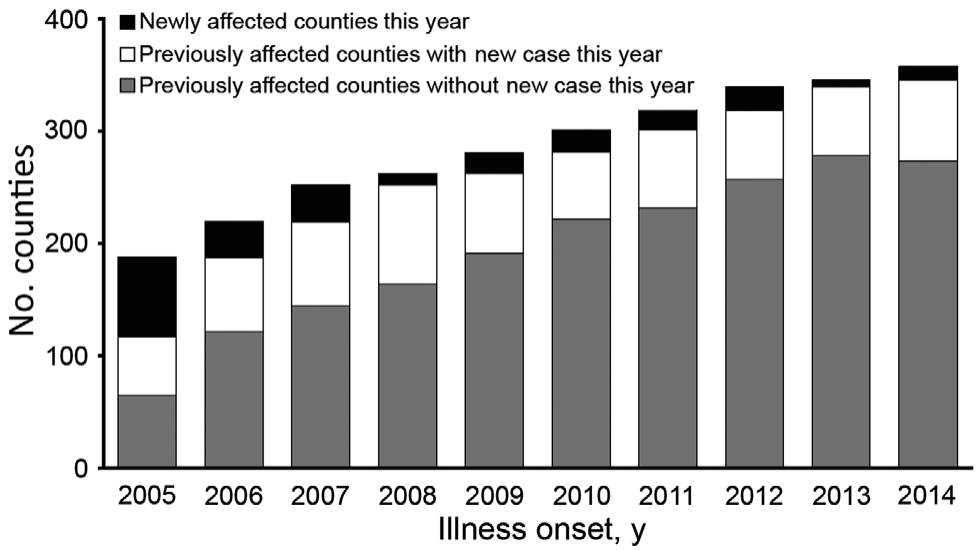
Trends in number of counties affected by probable and confirmed human anthrax, China, 2005–2014.

The cumulative number of counties affected by probable or confirmed human anthrax continued to increase from 188 (6.1% of the 3,074 counties in China) in 2005 to 358 (11.6%) in 2014. During the same period, although 52–88 previously affected counties reported new probable or confirmed human anthrax cases every year, newly affected counties were continuously reported (range 6–71 cases each year) (Figure 4). In contrast, the number of counties reporting confirmed human anthrax cases was much smaller compared with the number of counties affected by probable and confirmed cases. However, the pattern was similar and the cumulative number of affected counties with confirmed human anthrax increased from 41 in 2005 to 104 in 2014 (Technical Appendix Figure 1).

**Figure 5:**
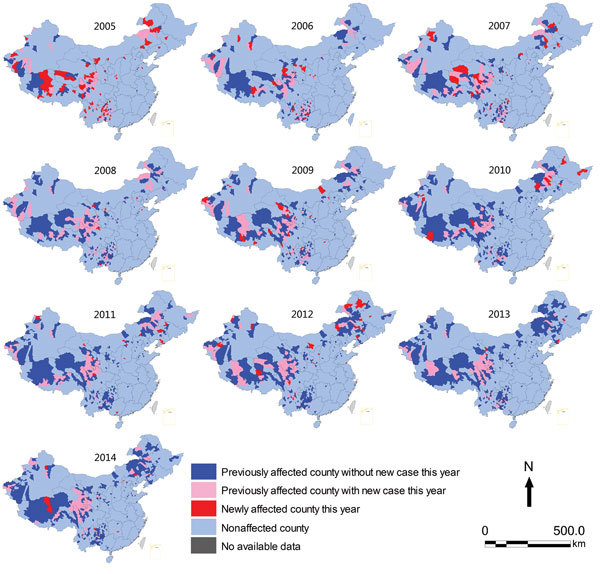
Geographic distribution of counties affected by probable and confirmed human anthrax, China, 2005–2014.

The counties affected by probable and confirmed human anthrax were located mainly in southwestern and northeastern China. Most newly affected counties were adjacent to previously affected counties. However, there were also newly affected counties not adjacent to previously affected counties in eastern China, such as counties in Shandong, Jiangsu, and Hunan Provinces (Figure 5). The pattern of geographic distribution in affected counties was similar when only confirmed human anthrax cases were included in the analysis, except that there were fewer counties with confirmed cases (Technical Appendix Figure 2).

### Demographic Characteristics

During 2005–2014, a total of 86.7% of human anthrax cases were found in farmers and herdsmen. Rural cases accounted for 92.4% of all cases (Table). The overall male:female case ratio was 2.8:1, and there was no obvious changing trend during this period. The proportion of urban cases fluctuated around 7%, except for 15% in 2011. In rural areas, younger persons, usually men <34 years of age, were more commonly affected. In urban areas, persons >40 years of age were more commonly affected. For affected persons <1–14 and >65 y of age, cases were more common in female patients than in male patients (Technical Appendix Figure 3).

**Table:** Demographic and diagnostic characteristics of patients with human anthrax, by diagnosis type, China, 2005–2014*

Characteristic	Probable cases, n = 3,121	Confirmed cases, n = 258	Total, n = 3,379
Sex			
M	2,273 (72.8)	210 (81.4)	2,483 (73.5)
F	848 (27.2)	48 (18.6)	896 (26.5)
Median age, y, (IQR)	38 (27–49)	40 (30–49)	38 (27–49)
Age group, y			
0–14	229 (7.3)	11 (4.3)	240 (7.1)
15–19	158 (5.1)	8 (3.1)	166 (4.9)
20–24	230 (7.4)	20 (7.8)	250 (7.4)
25–29	309 (9.9)	25 (9.7)	334 (9.9)
30–34	352 (11.3)	24 (9.3)	376 (11.1)
35–39	415 (13.3)	34 (13.2)	449 (13.3)
40–44	379 (12.1)	39 (15.1)	418 (12.4)
45–49	282 (9.0)	38 (14.7)	320 (9.5)
50–54	212 (6.8)	18 (7.0)	230 (6.8)
55–59	201 (6.4)	22 (8.5)	223 (6.6)
60–64	167 (5.4)	11 (4.3)	178 (5.3)
>65	187 (6.0)	8 (3.1)	195 (5.8)
Occupation			
Farmer or herdsman	2,700 (86.5)	231 (89.5)	2,931 (86.7)
Infants or students†	248 (7.9)	12 (4.7)	260 (7.7)
Other‡	173 (5.5)	15 (5.8)	188 (5.6)
Rural residence§	2,889 (92.6)	234 (90.7)	3,123 (92.4)
Fatal outcome	36 (1.2)	5 (1.9)	41 (1.2)
Clinical forms			
Cutaneous	3,055 (97.9)	252 (97.7)	3,307 (97.9)
Inhalational	0	1 (0.4)	1 (0.0)
Gastrointestinal	5 (0.2)	1 (0.4)	6 (0.2)
Unknown¶	61 (2.0)	4 (1.6)	65 (1.9)
Median onset to diagnosis interval, d (IQR)	4.0 (2.0–6.7)	5.0 (3.0–8.3)	4.0 (2.4–7.0)
Year of illness onset			
2005	497 (15.9)	38 (14.7)	535 (15.8)
2006	417 (13.4)	35 (13.6)	452 (13.4)
2007	400 (12.8)	26 (10.1)	426 (12.6)
2008	309 (9.9)	29 (11.2)	338 (10.0)
2009	334 (10.7)	17 (6.6)	351 (10.4)
2010	265 (8.5)	25 (9.7)	290 (8.6)
2011	279 (8.9)	30 (11.6)	309 (9.1)
2012	220 (7.1)	17 (6.6)	237 (7.0)
2013	181 (5.8)	12 (4.7)	193 (5.7)
2014	219 (7.0)	29 (11.2)	248 (7.3)

*Values are no. (%) unless otherwise indicated. Percentages may not total 100 because of rounding. IQR, interquartile range. †Infants include children attending and not attending kindergarten. Students include primary, secondary, and college students. ‡Includes teacher, laborers, self-employed and unemployed, workers, food industry personnel, retired persons, and cadres of staff. §If the residence community was connected to seats of county or municipal government through public facilities, residence facilities and other facilities, or mine area, development zone, research institutes, higher education establishments, farming communities or tree farming communities with >3,000 of permanent residents, it was considered an urban residence. Otherwise, the community was considered a rural residence. ¶No information was available.

### Diagnostics

During 2005–2014, a total of 3,379 human anthrax cases were reported, of which 257 (7.6%) were confirmed cases (Table). The proportion of confirmed cases fluctuated over this period, ranging from 4.8% in 2009 to 11.7% in 2014, and also varied between provinces; the highest was 36.4% in Shanxi and 29.5% in Inner Mongolia, and the lowest was 0% in Jiangsu, Shandong, and Hunan (Technical Appendix Figures 4, 5). During the same period, 97.7% of national probable and confirmed cases were cutaneous anthrax, which also accounted for most anthrax cases across all provinces (Technical Appendix Figure 6).

A total of 41 deaths were caused by anthrax; the proportion of fatal cases was 1.9% for confirmed cases and 1.2% for probable cases. The median time from illness onset to diagnosis was 4.0 days (interquartile range [IQR] 2.4–7.0 days), from diagnosis to death, 0 days (IQR 0–1.0 days), and from illness onset to death, 5 days (IQR 3.0–7.0 days). The median time from illness onset to diagnosis was 4.0 days (IQR 2.0–6.7 days) for persons with probable cases and 5.0 days (IQR 3.0–8.3 d) for persons with confirmed cases.

## Discussion

We conducted a systematic study of the epidemiology of human anthrax in China during 1955–2014. We believe that this study was useful because of recent epidemiologic changes and rapid socioeconomic changes during the past few decades. This study showed that, since 1990, the incidence rate and case-fatality rate for human anthrax has continued to decrease; only several hundred cases have been reported in the past 10 years. Most cases were in western China and peaked in August, but cases were reported infrequently in eastern China. This study also showed that cutaneous anthrax accounted for 98% of cases, and the largest proportion were in farmers and herdsmen. A low percentage of cases were laboratory confirmed.

The 3 historical peaks for human anthrax all occurred before 1980, after which a general decrease in cases occurred. Before 1980, some cases occurred in fur-processing workers infected by industrial exposure. From the 1960s onward, after implementation of strict quarantine and sanitary measures for animal fur and wool and improved industrial working conditions, the situation gradually improved to the point that after 1980 almost no human anthrax cases were seen in large cities, such as Beijing, Shanghai, and Tianjin. Since then, most anthrax cases have been caused by agricultural exposures. The fact that most anthrax cases were in male farmers or herdsmen further supports this feature of agricultural anthrax in China.

Cattle and sheep were and are the major infection source for human anthrax (*2*). The number of sheep slaughtered annually in China has increased from 42.419 million in 1980 to 270.995 million in 2012. The number of cattle slaughtered annually has increased from 3.322 million in 1980 to 48.281 million in 2013 (*10*).

Despite rapid increases in cattle and sheep populations, the case-fatality rate for human anthrax showed a continuous decrease, which suggested some success in control and prevention of this disease in livestock. Practices contributing to this success include timely reporting and detection of anthrax; rapid response to outbreaks and individual cases, which often includes restricting movement of livestock and related products from affected areas, tracing previous sources of possibly infectious livestock, and accordingly alerting related areas and departments; vaccination for possibly affected healthy livestock and related personnel, such as herdsmen, transportation staff, and slaughterhouse workers; prompt disposal of dead animals, bedding, and contaminated materials, for which a guide has been issued that specifies requirements for sites of incineration and burial of livestock carcasses, type of sanitizer and frequency of its use for livestock facilities and equipment, and general hygiene by persons who have contact with diseased or dead animals (*8*).

However, the present pattern of human cases clearly shows that any further improvement can be achieved only by using a proactive approach to the disease in livestock. This approach includes annual vaccination where outbreaks persist; improved laboratory diagnosis and participation, especially by veterinary diagnostic laboratories; genomic strain identification and mapping; and investigation of anomalous outbreaks (e.g., anthrax is normally a summer disease and outbreaks at other times are more characteristic of contaminated livestock feed). The reason for the decrease in the case-fatality rate for anthrax before 1980 was that this disease was largely industry related and was relatively easy to detect. With improvements in medical care, the incidence of anthrax showed a general downward trend, but our study showed that, since the 1980s, agricultural anthrax in rural areas has become a serious problem. Because of lack of convenient access to healthcare facilities and awareness of the need to seek early treatment for rural residence, the reported case-fatality rate increased and exceeded the rate during 1950s soon after the People’s Republic of China was established. However, one cannot presume that rural anthrax was absent during the earlier period. It is more likely that this disease was not reported, although cases were probably occurring at a level similar to that during the later period of reporting. In 1989, the case-fatality rate reached a high of 13.0%, which was probably caused by a major outbreak of intestinal anthrax in the Changdu District of Tibet (*11*).

From 1990 onward, a strengthened surveillance program was initiated in western China, and early detection of anthrax facilitated early treatment. This program contributed to a decrease in the case-fatality rate. This rate has decreased to <1% in recent years, which is consistent with the report that the case-fatality rate for cutaneous anthrax is now <1% after treatment (*12*). However, gastrointestinal anthrax has a higher risk for death, which can occur quickly, and this finding would preempt any clinical observations or sampling. These findings might explain the unusually high number of cutaneous cases relative to each gastrointestinal case.

Anthrax has not been eradicated from previously disease-endemic areas in China, and geographic distribution of the disease tends to expand into new areas. Each year, previously affected counties still accounted for a predominant proportion of counties with reports of cases that year. This finding could be caused by strong resistance of *B. anthracis* spores to environmental conditions and persistence of spores in old foci for disease (*13*). However, some provinces, such as Henan, which reported many anthrax cases in the past, has not reported any anthrax cases for >10 years, shows that it is possible to control anthrax.

Conversely, newly affected counties were reported every year during the study. Newly affected counties were generally adjacent to previously affected counties, which indicated a higher risk for importing anthrax from neighboring disease-endemic counties through contaminated livestock feed or movement of animals with latent infections. However, a few counties in Hunan and Jiangsu Provinces in eastern China, which are not near disease-endemic areas, also became newly affected in recent years. Investigations of these incidents showed that they were caused by long-distance transportation of infected livestock from disease-endemic areas. These investigations indicated the need for livestock inspection and ensuring that such livestock have been vaccinated >7–10 days before shipment.

As disease control improves, disease reporting also improves, and cases that would have been missed are now detected and reported. However, absence of disease reports is not the same as absence of disease. This situation indicates that provinces and large cities that have not had any anthrax cases in recent years are at risk for reemergence of anthrax. Humans get infected from animals with anthrax, and not vice versa. Therefore, humans will only be safe from this disease when it has been eradicated from livestock. Although eradication will require diligent surveillance and monitoring by the agricultural and veterinary communities, it has been achieved in many countries (*5*).

Our study had 2 limitations. First, we used data obtained through a passive surveillance system that was based on human health facilities nationwide. Thus, disease burden was probably underestimated because of various reasons, such as not seeking medical care. However, the clinical presentation for cutaneous anthrax makes patients more likely to seek medical care. Second, probable cases without laboratory confirmation of infection are also included in the analysis. Most cases were cutaneous, and their clinical manifestations are easily identified by medical personnel.

Laboratory diagnosis (based on isolation of *B. anthracis*) is not completely reliable. However, *B. anthracis* is susceptible to many antimicrobial drugs, and treatment of this type is commonly used. Such treatment could result in lesions being negative for *B. anthracis*. Although in recent years ELISA and PCR could be used for diagnosing confirmed cases, both techniques were rarely used at the local level because there were no reliable commercial kits for detecting antibodies against *B. anthracis* and no unified standard procedure for PCR of *B. anthracis* is currently available.

Technical Appendix Additional information for epidemiology of human anthrax in China, 1955−2014.

## References

[1] Woods CW, Ospanov K, Myrzabekov A, Favorov M, Plikaytis B, Ashford DA. Risk factors for human anthrax among contacts of anthrax-infected livestock in Kazakhstan. Am J Trop Med Hyg. 2004;71:48–52. 15238688

[2] World Health Organization. Anthrax in humans and animals. 4th ed. Geneva: The Organization; 2008. 26269867

[3] Swartz MN. Recognition and management of anthrax: an update. N Engl J Med. 2001;345:1621–6. 10.1056/NEJMra012892 11704686

[4] Abbara A, Brooks T, Taylor GP, Nolan M, Donaldson H, Manikon M, et al. Lessons for control of heroin-associated anthrax in Europe from 2009–2010 outbreak case studies, London, UK. Emerg Infect Dis. 2014;20:1115–22. 24959910 10.3201/eid2007.131764 PMC4073855

[5] Hugh-Jones M. 1996–97 global anthrax report. J Appl Microbiol. 1999;87:189–91. 10.1046/j.1365-2672.1999.00867.x 10475945

[6] Klietmann WF, Ruoff KL. Bioterrorism: implications for the clinical microbiologist. Clin Microbiol Rev. 2001;14:364–81. 10.1128/CMR.14.2.364-381.2001 11292643 PMC88979

[7] Jernigan JA, Stephens DS, Ashford DA, Omenaca C, Topiel MS, Galbraith M, et al.; Anthrax Bioterrorism Investigation Team. Bioterrorism-related inhalational anthrax: the first 10 cases reported in the United States. Emerg Infect Dis. 2001;7:933–44. 10.3201/eid0706.010604 11747719 PMC2631903

[8] Dong SL. Progress in the control and research of anthrax in China. Presented at: International Workshop on Anthrax; 1989 Apr 11–13; Winchester, UK.

[9] Chinese Ministry of Health. National surveillance protocol for anthrax [in Chinese] [cited 2015 May 13]. http://www.chinacdc.cn/jkzt/crb/tj/tjjc/200608/W020130110372314597578.pdf

[10] National Bureau of Statistics of China. National annual statistics dataset [in Chinese] [cited 2015 Apr 6]. http://data.stats.gov.cn/workspace/index?m=hgnd

[11] Li AF, Zhang XG, Jiang HZ, Liang XD. First detection of laryngeal anthrax’s epidemic [in Chinese]. Chin J Zoonoses. 1992;8:19.

[12] Dixon TC, Meselson M, Guillemin J, Hanna PC. Anthrax. N Engl J Med. 1999;341:815–26. 10.1056/NEJM199909093411107 10477781

[13] Manchee RJ, Broster MG, Stagg AJ, Hibbs SE. Formaldehyde solution effectively inactivates spores of *Bacillus anthracis* on the Scottish island of Gruinard. Appl Environ Microbiol. 1994;60:4167–71. 16349444 10.1128/aem.60.11.4167-4171.1994 PMC201953

