# Supplementary material for: Epidemiology of Human Anthrax in China, 1955−2014
Source: Emerg Infect Dis. 2017 Jan;23(1):14–21. doi: 10.3201/eid2301.150947 (PMC5176222; doi:10.3201/eid2301.150947)
Supplement: Technical Appendix — Additional information for epidemiology of human anthrax in China, 1955−2014. [file 15-0947-Techapp-s1.pdf]

Article DOI: http://dx.doi.org/10.3201/eid2301.150947

Epidemiology of Human Anthrax in China, 1955–2014

Technical Appendix. Additional information for epidemiology of human anthrax in China, 1955–2014

Technical Appendix Table. Diagnostic criteria for human anthrax in China, 1955–2014*

	Period		
Case category	1955 –1998 Aug 31	1998 Sep 1–2008 Aug 31	2008 Sep 1–2014
Suspected	Clinical manifestation†	Typical clinical manifestation of cutaneous anthrax, or clinical manifestation of other anthrax and epidemiologic history‡	Clinical manifestation and epidemiologic history
Probable	Clinical manifestation and epidemiologic history	Clinical manifestation and microscopic examination	Clinical manifestation and microscopic examination
Confirmed	Clinical manifestation and microscopic examination/bacteria isolation	Clinical manifestation and bacteria isolation/antibody increase	Clinical manifestation and bacteria isolation/antibody increase

*A positive laboratory test result was 1) square-ended, gram-positive, rod-shaped, large bacteria in chains found by microscopic examination of a clinical specimen, such as excreta of skin ulcer, sputum, feces, blood, or cerebrospinal fluid; 2) a positive result for isolation of *Bacillus anthracis*; or 3) a $\geq$4-fold increase in specific antibody titers against *B. anthracis* between acute-phase and convalescent-phase serum samples.

†1) Erythema, papules, and blisters of unknown cause on localized skin of exposed areas, such as the face, neck, hands, or forearms. Swelling and infiltration around the tissue, followed by ulcerative black eschar without pain, surrounded by rubefaction and swelling. Swelling and often festering at a draining lymph node, concurrent with fever, headache, and arthralgia. Massive edema and necrosis could occur at the localized area for a small proportion of persons with severe cases. 2) Sudden onset of fever, abdominal distension, serious abdominal pain, diarrhea (often with bloody stool or watery bloody stool). Nausea and vomiting might be present and contain blood streaks and bile. Symptoms and signs for systems other than digestive tract might be present. 3) High fever, dyspnea, chest pain and cough, and thick bloody sputum. Often a fine moist rale might be the only pulmonary sign present. Widened mediastinal shadow on a chest radiograph. Pleural effusion could often be found. 4) Serious headache, vomiting, and stiff neck, followed by deliration, coma, and respiratory failure, often with bloody cerebrospinal fluid. All signs and symptoms could occur after clinical manifestations 1–3 and could also occur directly. 5) Signs and symptoms of systemic poisoning, high fever, shivering, septic shock and disseminated intravascular coagulation, petechiae or massive ecchymosis on the skin, active bleeding from all orifices, rapidly followed by circulatory and respiratory failure. Numerous gram-positive, rod-shaped, large bacteria found by microscopic examination. All could occur after clinical manifestations 1–3 and could also occur directly.

‡Epidemiologic history included 1) living in areas with reports of confirmed anthrax or traveling to such places within 14 d of disease onset; or 2) having an occupation, such as fur processing and farming, in an area possibly contaminated with anthrax spores, which are likely to result in exposure to anthrax; contact with suspected sick/dead animals or their remains, and consumption of suspected sick/dead animals or their food products.

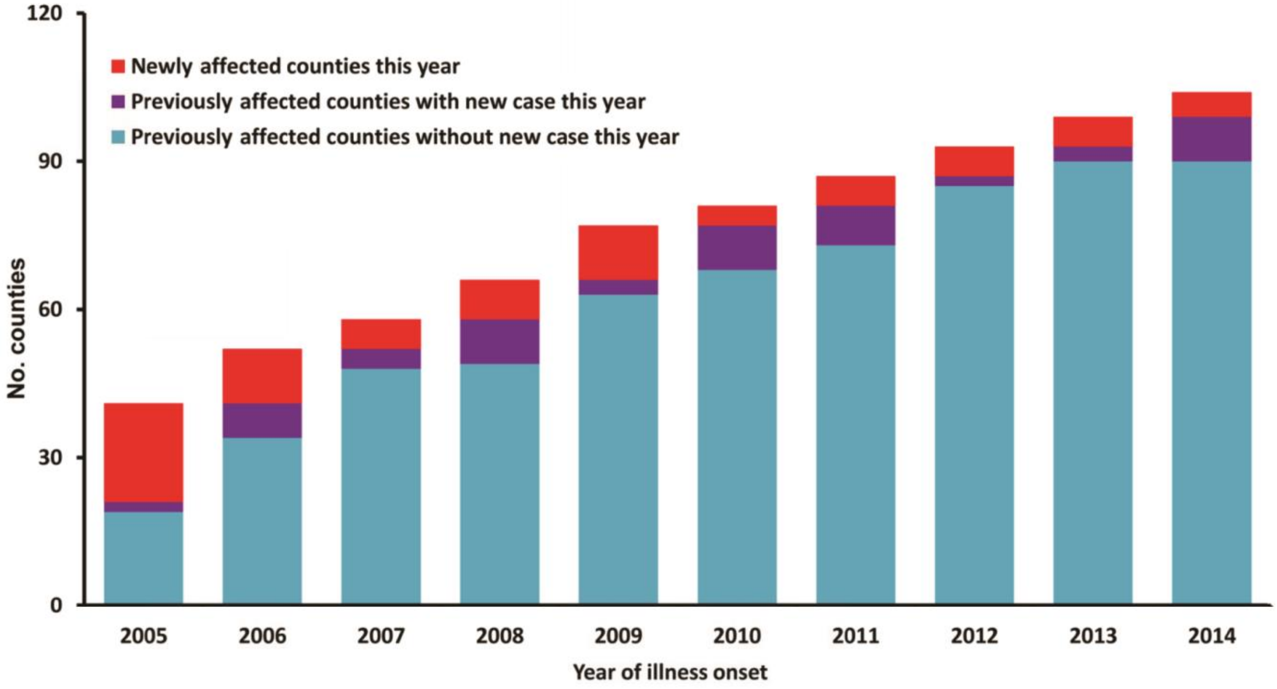

Technical Appendix Figure 1. Trends in number of counties with confirmed human anthrax, China, 2005–2014.

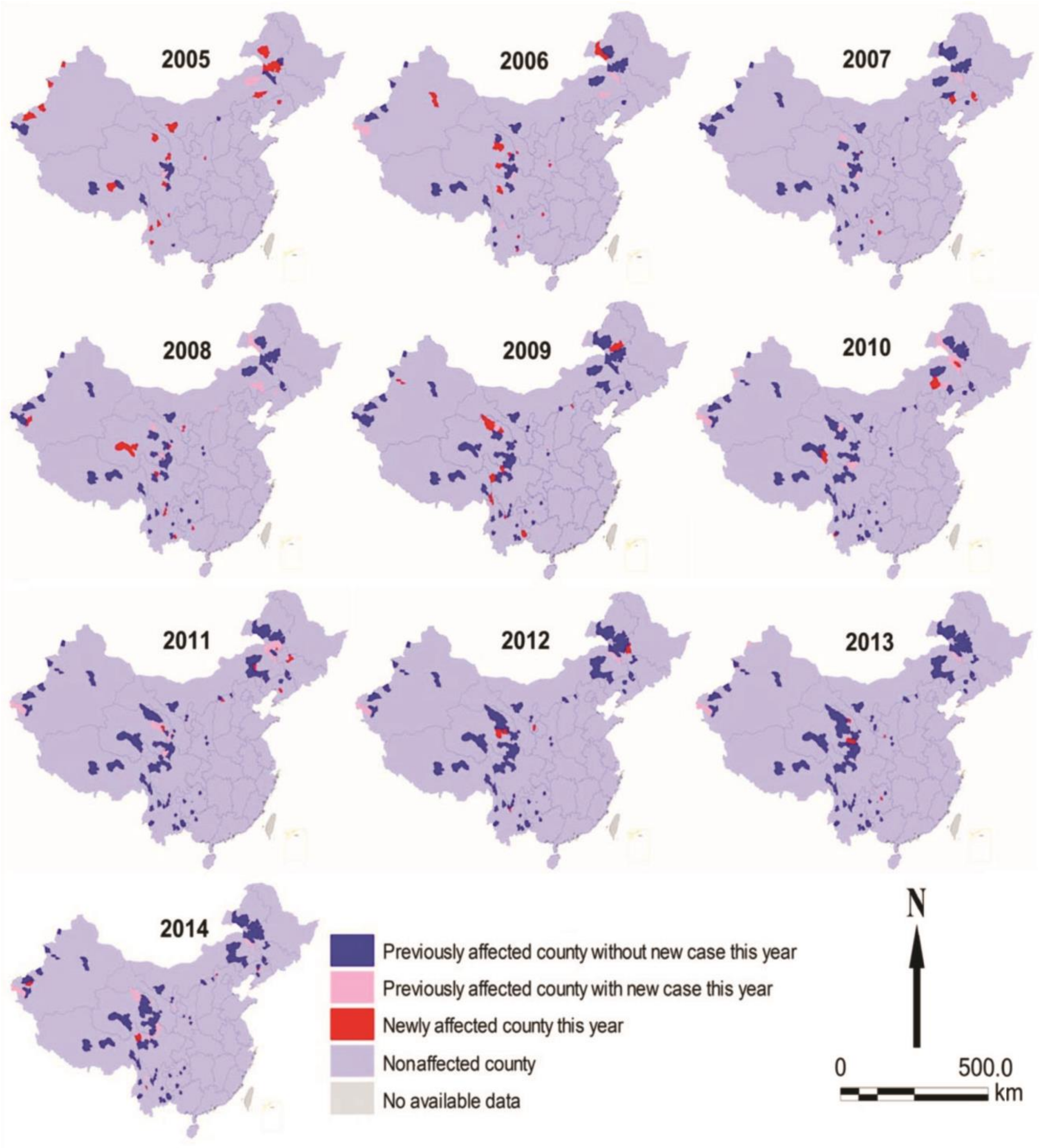

Technical Appendix Figure 2. Geographic distribution of counties with confirmed human anthrax, China, 2005–2014.

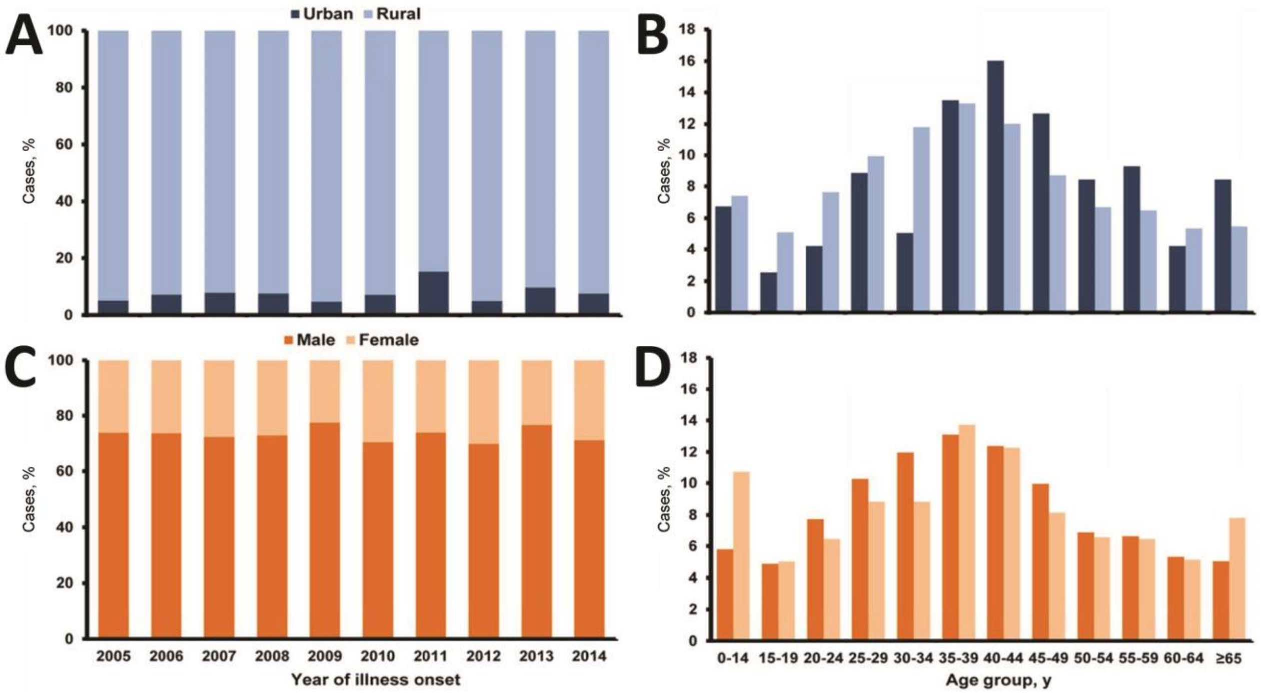

Technical Appendix Figure 3. Demographic characteristics of probable and confirmed human anthrax cases by sex, residence type, and year, China, 2005–2014. A) Proportion of rural and urban cases by year. B) Age distribution of cases by residence type. C) Proportion of male and female cases by year. D) Age distribution of cases by sex.

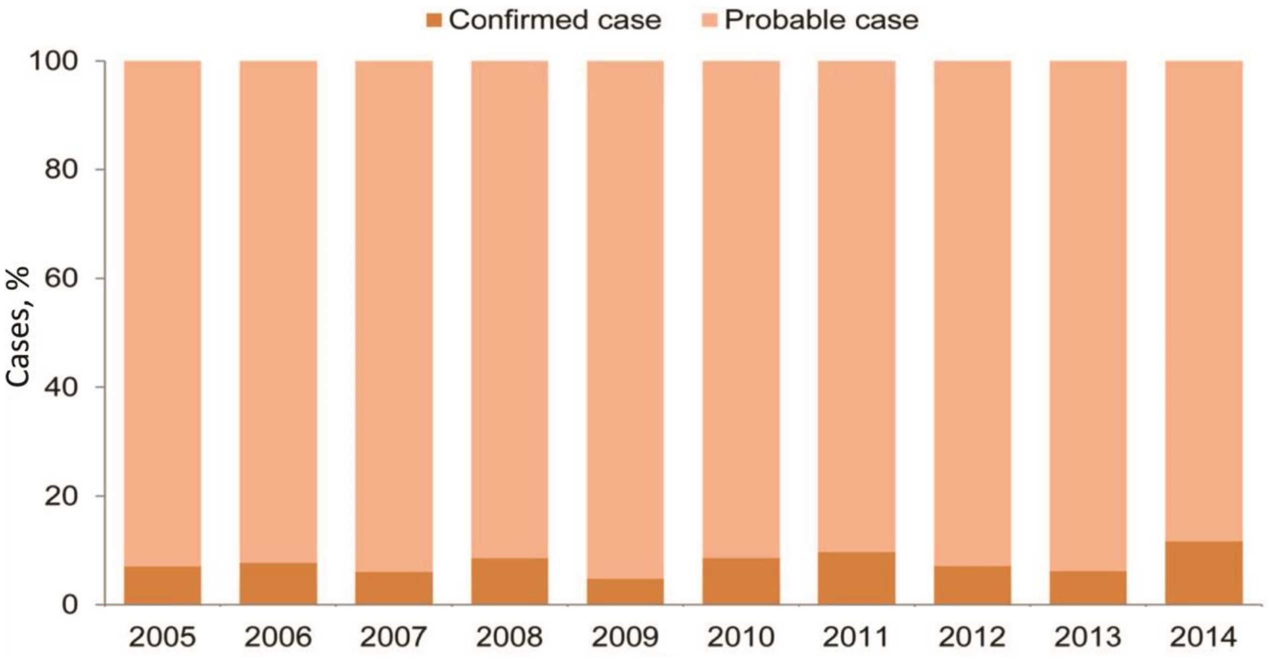

Technical Appendix Figure 4. Trends in proportion of probable and confirmed human anthrax cases, China, 2005–2014.

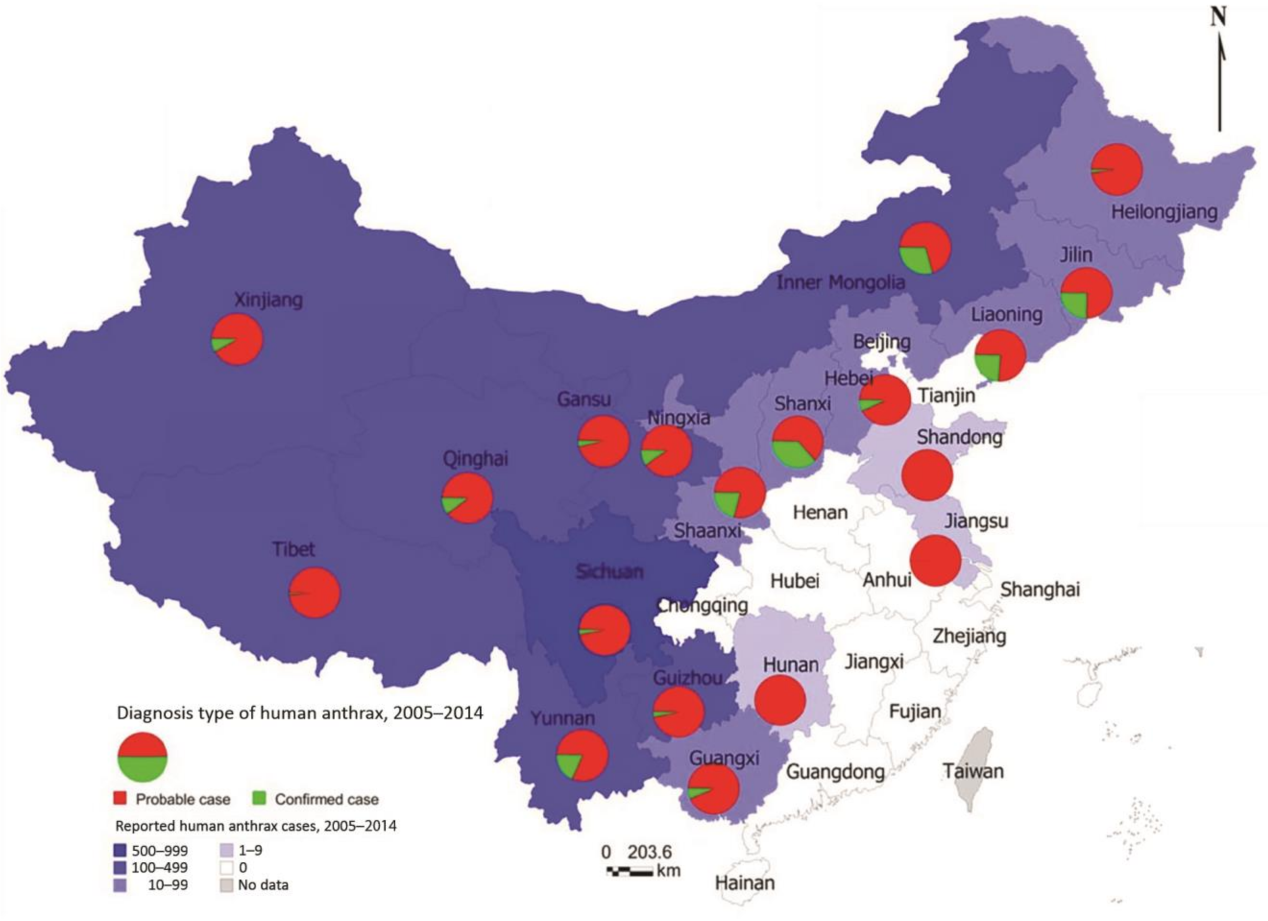

Technical Appendix Figure 5. Provincial distribution of human anthrax cases by diagnosis type, China, 2005–2014.

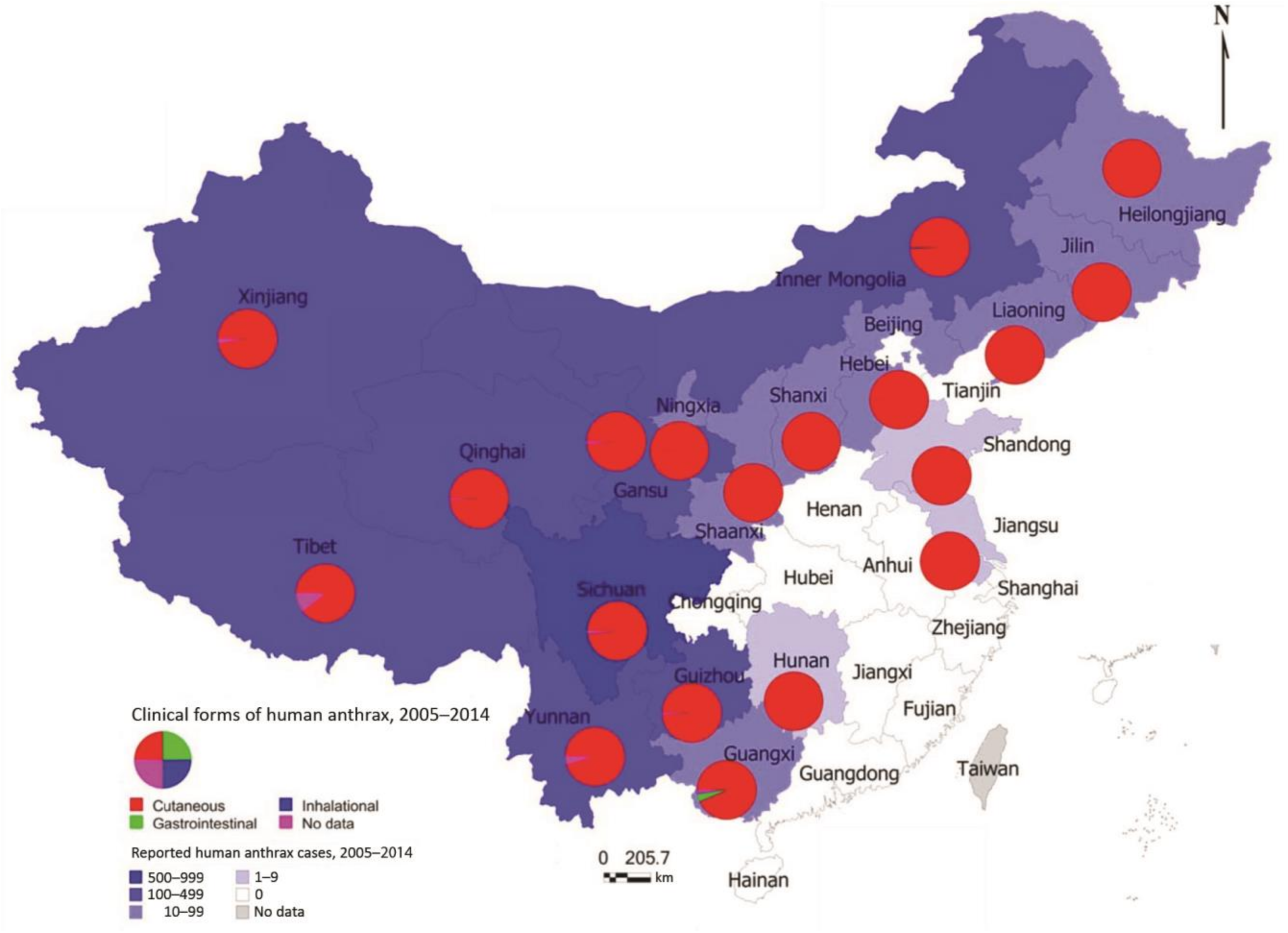

Technical Appendix Figure 6. Provincial distribution of clinical presentation for human anthrax cases, China, 2005–2014.
